# Mycorrhizal Fungus-Based Fertilizer Mitigates Salt Stress Damage on Colored-Fiber Cotton in Semi-Arid Salinized Soil of Brazil

**DOI:** 10.3390/plants15213247

**Published:** 2026-10-22

**Authors:** Evanilson Souza de Almeida, Jackson Silva Nóbrega, Geovani Soares de Lima, Lauriane Almeida dos Anjos Soares, Valéria Fernandes de Oliveira Sousa, Weslley Bruno Belo de Souza, Valeska Karolini Nunes Oliveira, Paulo Vinicius de Oliveira Freire, Reynaldo Teodoro de Fátima, Francisco Romário Andrade Figueiredo, Tailson Andrade Sampaio, José Ramon Tomaz de Aquino Alves, Hans Raj Gheyi, Francisco de Assis da Silva, André Alisson Rodrigues da Silva, Antônio Gustavo de Luna Souto, Toshik Iarley da Silva

**Affiliations:** 1 Graduate Program in Agricultural Engineering, Federal University of Campina Grande, Campina Grande 58429-900, PB, Brazil; evanilsom46@gmail.com (E.S.d.A.); weslleybruno96@hotmail.com (W.B.B.d.S.); valeska.karoline2015@gmail.com (V.K.N.O.); hgheyi@gmail.com (H.R.G.); 2 Rurópolis University Campus, Federal University of Western Pará, Rurópolis 68165-000, PA, Brazil; 3 Academic Unit of Agricultural Sciences, Federal University of Campina Grande, Pombal 58840-000, PB, Brazil; geovani.soares@professor.ufcg.edu.br (G.S.d.L.); lauriane.soares@professor.ufcg.edu.br (L.A.d.A.S.); valeriafernandesbds@gmail.com (V.F.d.O.S.); oliveiravinicius5370@gmail.com (P.V.d.O.F.); reynaldoteodoro@outlook.com (R.T.d.F.); tailsonandrade65@gmail.com (T.A.S.); josetomazjr5@gmail.com (J.R.T.d.A.A.); 4 Department of Agronomic and Forest Sciences, Federal Rural University of the Semi-Arid, Mossoró 59625-000, RN, Brazil; romarioagroecologia@yahoo.com.br; 5 Monte Alegre University Campus, Federal University of Western Pará, Monte Alegre 68220-000, PA, Brazil; agrofdsilva@gmail.com; 6 Juruti University Campus, Federal University of Western Pará, Juruti 68170-000, PA, Brazil; andrealisson_cgpb@hotmail.com; 7 Graduate Program in Agronomy, Federal University of Paraíba, Areia 58397-900, PB, Brazil; gusluso@hotmail.com; 8 Center for Agricultural, Environmental, and Biological Sciences, Federal University of Recôncavo of Bahia, Cruz das Almas 44380-000, BA, Brazil; iarley.toshik@gmail.com

**Keywords:** *Gossypium hirsutum* L., soil electrical conductivity, salt stress, *Rhizophagus irregularis*

## Abstract

In Northeastern Brazil, agricultural cultivation can be limited by excess soil salinity, which poses a challenge to crop production. To mitigate the impacts of salt stress on plants, it is necessary to develop appropriate technologies; the use of organomineral fertilizers based on biological inputs—such as mycorrhizal fungi—stands out among these. The objective of this study was to evaluate the growth, gas exchange, photosynthetic pigment contents, and production components of colored-fiber cotton grown in salinized soil under concentrations of an organomineral fertilizer based on mycorrhizal fungi. The experiment was carried out under field conditions at the Federal University of Campina Grande, Pombal campus, PB, Brazil, using a randomized block design, in a 5 × 4 factorial scheme, referring to five electrical conductivity of the saturation extract (ECse) levels (3.7, 4.9, 6.1, 7.3 and 8.5 dS m^−1^) and four concentrations of an organomineral fertilizer based on mycorrhizal fungi (FMF = 0—Control; 1.5, 2.5 and 4.5 g L^−1^). Soil salinity of 5.0 dS m^−1^ inhibited growth and gas exchange of colored-fiber cotton. The 2.7 g L^−1^ concentration improved water use efficiency in cotton plants up to 4.9 dS m^−1^. The application of up to 1.9 g L^−1^ increased photosynthetic pigment synthesis under ECse levels of up to 5.8 dS m^−1^. Application of FMF at 2.5 g L^−1^ increased cotton production at ECse levels up to 5.5 dS m^−1^. Under the experimental conditions of this study, the use of an organomineral fertilizer based on mycorrhizal fungi proves to be an efficient strategy for producers in arid and semi-arid regions, which are frequently affected by excess soil salinity.

## 1. Introduction

Cotton (*Gossypium hirsutum* L.) is one of the crops that stands out for its socioeconomic importance, serving as the main source of natural fiber and representing about 35% of the total fibers used worldwide [1]. The demand for naturally colored-fiber cotton has grown due to its environmental and economic benefits, eliminating the need for artificial dyeing [2].

In the 2025 harvest, Brazil produced approximately 9,920,078 tons of cotton in an area of 2,151,656 hectares. The Central–West region was responsible for about 77% of this production, followed by the Northeast region with approximately 22% [3], significantly contributing to the generation of employment and income. In addition, the Northeast region is recognized for producing some of the best cotton fibers in the world, thanks to favorable climatic conditions that favor production [4].

However, several factors can reduce the yield of colored cotton in the Northeast region of Brazil, such as water and/or soil salinity [5]. Excess salts in the soil impair the availability of water and nutrients to plants, leading to an increase in sodium in the soil and hampering the absorption of other ions such as calcium, phosphorus, and potassium [6,7].

In cotton, excess salts in the soil can compromise gas exchange and plant growth, leading to losses in fiber production and quality [8,9,10]. The harmful effects of salinity include a reduction in the initial absorption of water, which reduces leaf water potential and turgor pressure, generating osmotic stress, consequently resulting in ionic stress due to the excess of toxic ions in the leaves, and oxidative stress due to the accumulation of reactive oxygen species [11]. This condition negatively influences growth, such that smaller plants, with smaller leaf area and less root growth, in turn, transpire less water, assimilate less CO_2_ and consequently produce smaller quantities of assimilates for their growth [8]. This negatively compromises gas exchange and synthesis of photosynthetic pigments, increases cell membrane damage and limits the production of cotton [8,10].

A report on the status of salt-affected soils estimates that 1.381 billion hectares, or 10.7% of the world’s total land area, are currently salinized and notes that the global rise in temperature could increase these figures to between 24% and 32% of the Earth’s surface [12]. In this context, it is essential to identify strategies capable of mitigating the effects of soil salinity and enabling the cultivation of colored-fiber cotton in semi-arid areas. Among the promising alternatives, the use of mycorrhizal fungi stands out [13,14]. These fungi can increase nutrient and water absorption, maintain osmotic balance, stimulate antioxidant functions to mitigate damage caused by reactive oxygen species, elevate photosynthetic rate, and modulate hormone levels, decreasing the detrimental effects of salinity on plant growth and development [15].

Ahmadi-Nouraldinvand [16], when studying the effect of applying mycorrhizal fungus-based biofertilizer on wheat plants, observed that biofertilizer increased leaf water potential, soluble protein, soluble sugar, catalase, peroxidase, and polyphenol oxidase, playing an improvement role by promoting the accumulation of these osmolytes and the action of these antioxidant enzymes under saline conditions, which reduced oxidative stress in plants.

Despite this, results indicating the effect of using mycorrhizal fungi-based fertilizers on the cultivation of naturally colored fiber cotton in saline soil remain scarce, making it necessary to conduct research to demonstrate the viability of their use. Therefore, this study hypothesizes that a mycorrhizal fungus-based fertilizer may be capable of mitigating the deleterious effects of soil salinity stress by improving nutrient availability and increasing photosynthetic pigment synthesis and gas exchange in cotton plants, cv. BRS Jade. Thus, the objective was to evaluate the effects of applying different concentrations of mycorrhizal fungus-based fertilizer on the growth, gas exchange, chlorophyll content, and yield of colored-fiber cotton in salinized soil.

## 2. Results

### 2.1. Differential Analysis of Leaf Saturation Extract

The interaction between the levels of electrical conductivity of the soil saturation extract (ECse) and concentrations of organomineral fertilizer based on mycorrhizal fungi (FMF) had a significant effect on the relative water content, electrolyte leakage and leaf succulence of cotton plants, cv. BRS Jade, at 85 days after sowing (Table 1).

**Figure 1:**
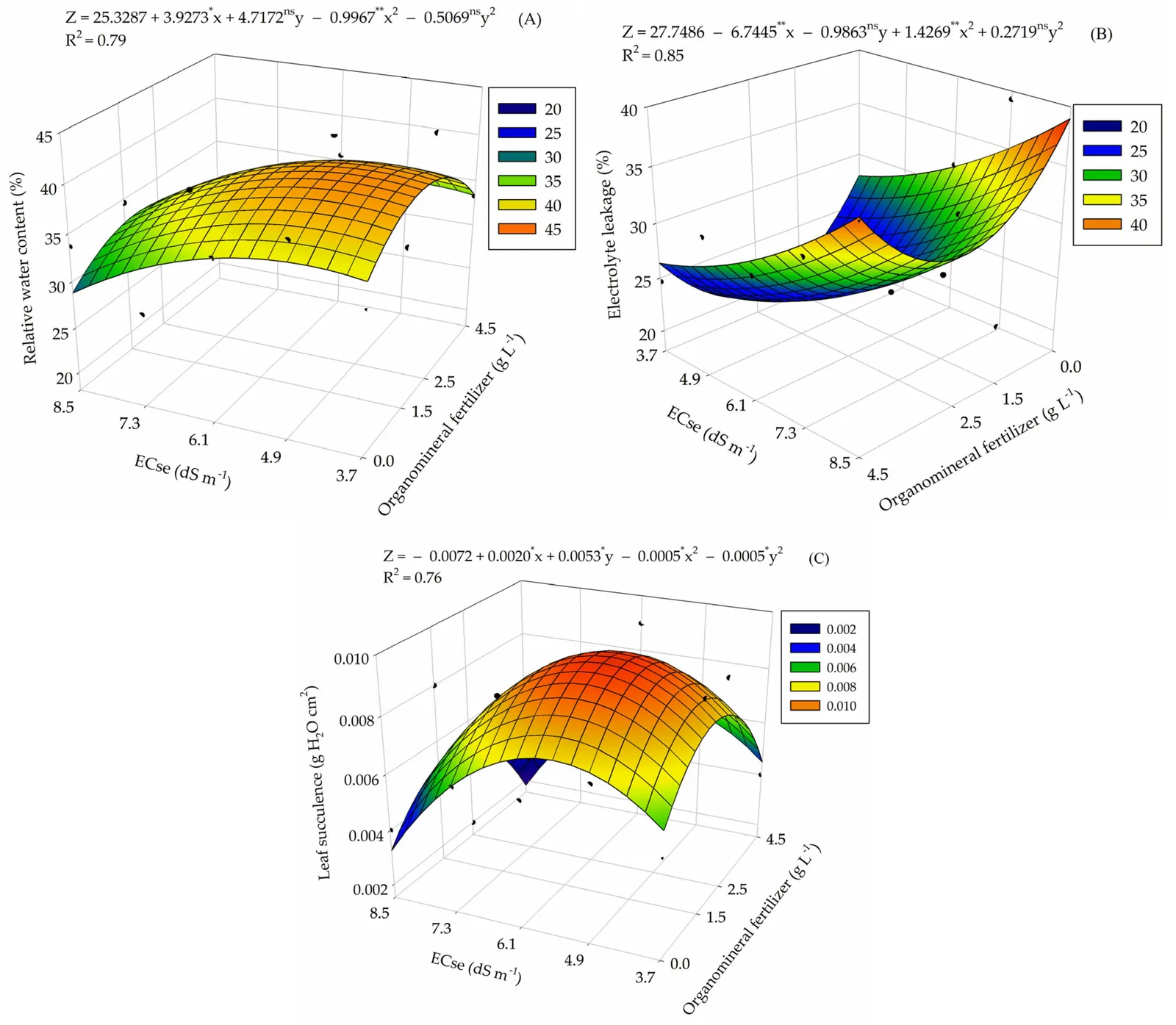
Relative water content—RWC (**A**), electrolyte leakage—EL (**B**), and leaf succulence—SUC (**C**) of cotton cv. BRS Jade, as a function of the levels of electrical conductivity of the saturation extract (ECse) and concentrations of organomineral fertilizer based on mycorrhizal fungi (FMF), at 85 days after sowing. (*) significant at 0.05 probability level; (**) significant at 0.01 probability level; (ns) not significant.

For relative water content (Figure 1A), the highest value (40.16%) was observed in plants under an ECse of 4.7 dS m^−1^ and an FMF concentration of 2.0 g L^−1^. However, the lowest value (26.27%) was reached in plants grown under an ECse of 8.5 dS m^−1^ and an FMF concentration of 4.5 g L^−1^.

Electrolyte leakage in the leaf blade was high (39.02%) in cotton plants grown under an ECse of 8.5 dS m^−1^ in the absence of FMF application. However, in plants subjected to the lowest level of ECse (3.7 dS m^−1^), FMF application at a concentration of 2.4 g L^−1^ resulted in a decrease in electrolyte leakage (19.85%) (Figure 1B).

Leaf succulence increased as a function of the ECse levels, with a maximum value of 0.0075 g H_2_O cm^2^ obtained under a saturation extract salinity of 5.0 dS m^−1^ and an FMF application of 2.0 g L^−1^. When comparing plants subjected to an ECse of 8.5 dS m^−1^ to those cultivated under 3.7 dS m^−1^ and in the absence of application, there was a reduction of 97% (Figure 1C).

### 2.2. Differential Analysis of Gas Exchange Related Traits

The interaction between water salinity levels and concentrations of organomineral fertilizer based on mycorrhizal fungi (FMF) (Table 2) significantly influenced (*p* ≤ 0.01) the gas exchange of cotton cv. BRS Jade at 85 days after sowing.

**Figure 2:**
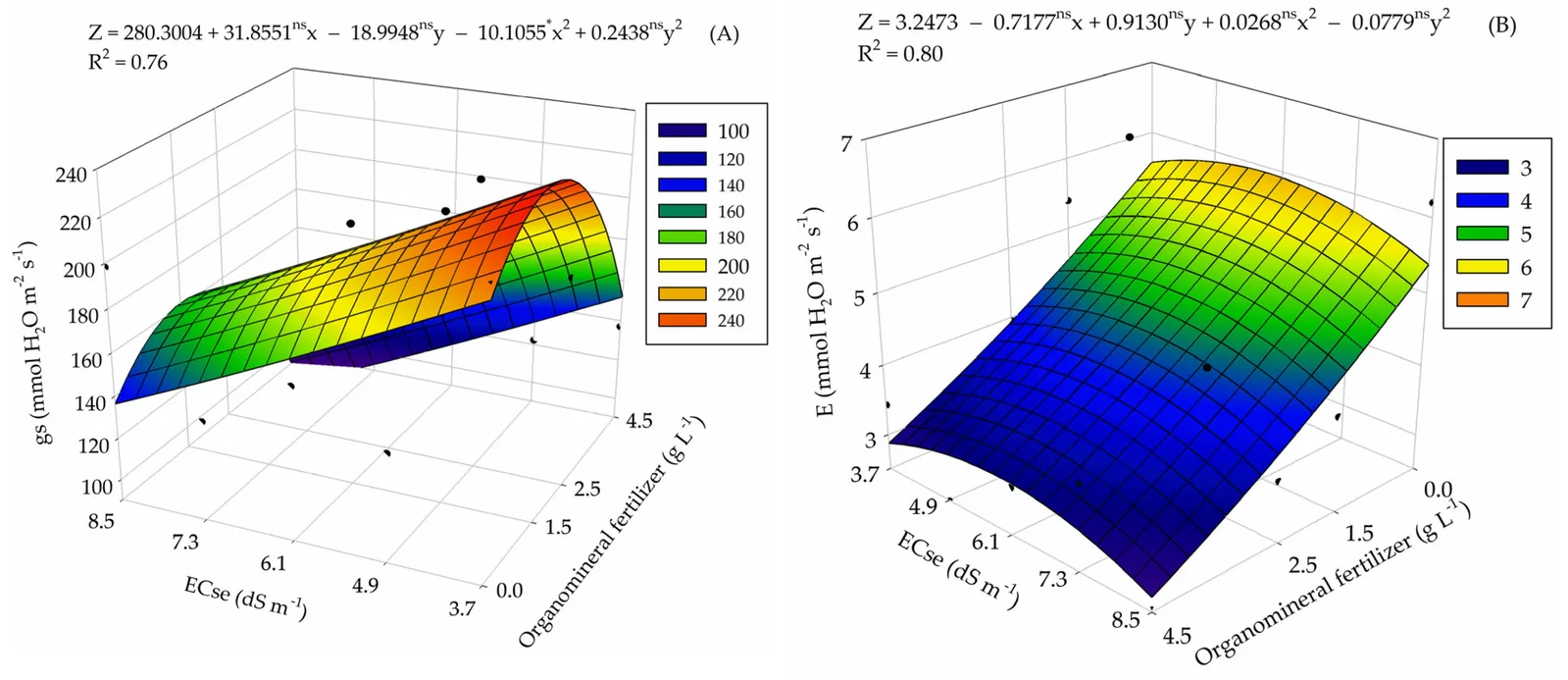

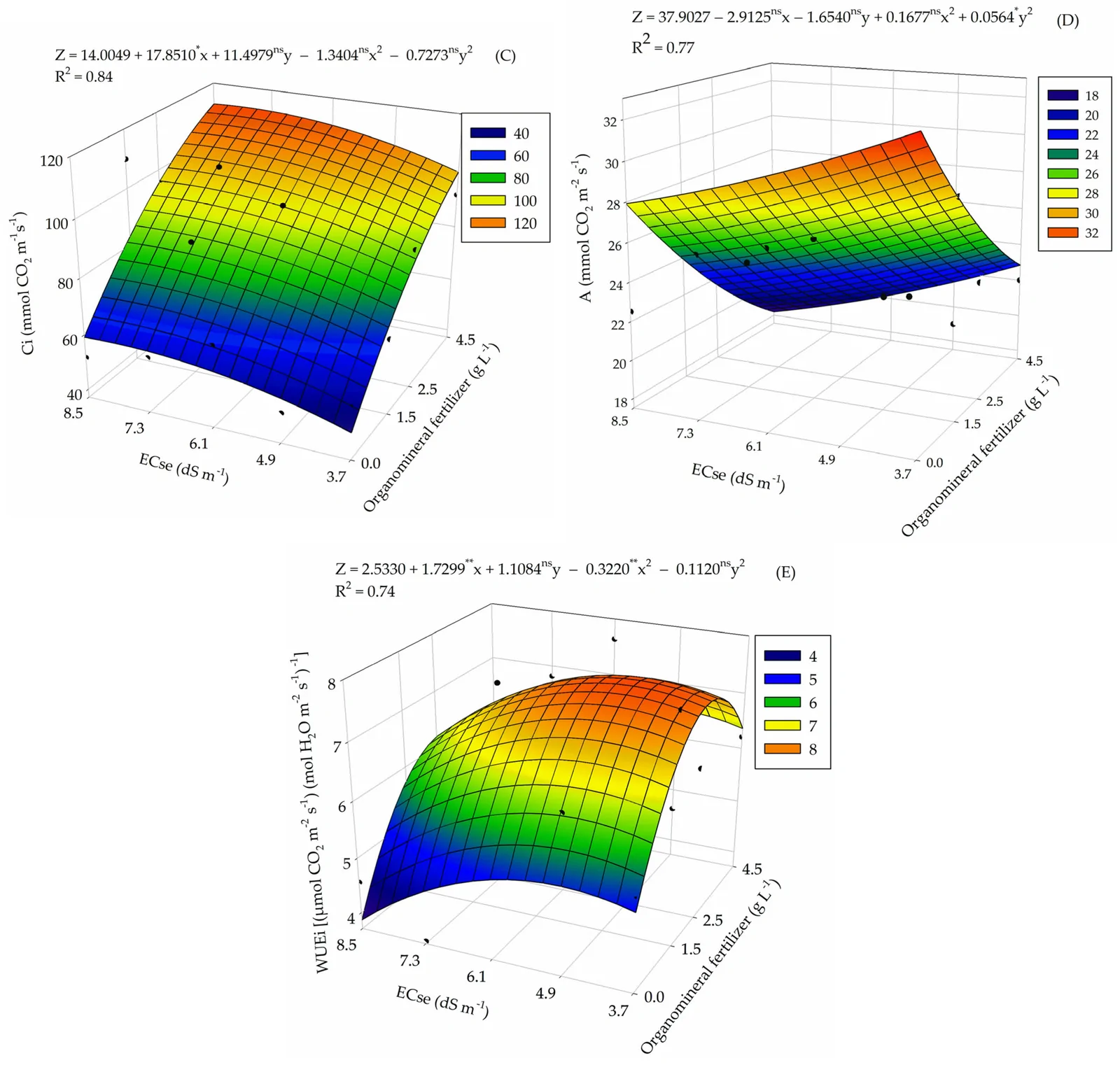
Stomatal conductance—*gs* (**A**), transpiration—*E* (**B**), internal CO_2_ concentration—*Ci* (**C**), CO_2_ assimilation rate—*A* (**D**), and instantaneous water use efficiency—*WUEi* (**E**) of cotton under electrical conductivities of the saturation extract (ECse) and concentrations of organomineral fertilizer based on mycorrhizal fungi (FMF). (*) significant at 0.05 probability level; (**) significant at 0.01 probability level; (ns) not significant.

Regarding stomatal conductance (Figure 2A), the maximum value (238.45 mmol H_2_O m^−2^ s^−1^) was obtained in plants under a soil saturation extract electrical conductivity of 3.7 dS m^−1^ and with an FMF application of 1.6 g L^−1^, which represents an increase of 11.76% compared to those cultivated under an ECse of 3.7 dS m^−1^ and an FMF concentration of 0 g L^−1^.

For transpiration (Figure 2B), a maximum estimated value of 5.91 mmol H_2_O m^−2^ s^−1^ was obtained in plants cultivated under an ECse of 5.9 dS m^−1^ and an FMF concentration of 0 g L^−1^. On the other hand, plants subjected to an ECse of 8.5 dS m^−1^ and an FMF concentration of 4.5 g L^−1^ reached a minimum estimated value of 2.68 mmol H_2_O m^−2^ s^−1^.

The salinity of the saturation extract increased the internal CO_2_ concentration (Figure 2C), and its maximum estimated value of 112.66 μmol mol^−1^ was obtained under an ECse of 7.9 dS m^−1^ and an FMF concentration of 4.5 g L^−1^. On the other hand, cotton cultivation in soil with an ECse of 3.7 dS m^−1^ and an FMF concentration of 0 g L^−1^ resulted in the lowest value of *Ci* (46.59 μmol mol^−1^).

Increments in salinity in the soil saturation extract and concentrations of organomineral fertilizer based on mycorrhizal fungi resulted in a decrease in the CO_2_ assimilation rate, from 32.54 to 18.18 μmol CO_2_ m^−2^ s^−1^ when comparing cotton plants grown under ECse levels of 3.7 and 8.5 dS m^−1^ and FMF concentrations of 0 and 4.5 g L^−1^, respectively (Figure 2D).

Water use efficiency increased in plants grown under an ECse of 4.9 dS m^−1^ with the application of FMF at a concentration of 2.7 g L^−1^, with a maximum value of 7.59 [(μmol CO_2_ m^−2^ s^−1^) (mol H_2_O m^−2^ s^−1^)^−1^] (Figure 2E). At this ECse level, there was a decrease in efficiency, with the lowest estimated value (3.89 [(μmol CO_2_ m^−2^ s^−1^) (mol H_2_O m^−2^ s^−1^)^−1^] in plants subjected to an ECse of 8.5 dS m^−1^ and an FMF concentration of 4.5 g L^−1^, representing a reduction of 49.20% compared to those cultivated under an ECse of 8.5 dS m^−1^.

### 2.3. Differential Analysis of Chlorophyll Contents

The interaction between the levels of electrical conductivity of the soil saturation extract (ECse) and the concentrations of organomineral fertilizer based on mycorrhizal fungi (FMF) had a significant effect on the contents of photosynthetic pigments, represented by the chlorophyll *a*, chlorophyll *b* and total chlorophyll indices, indicating that both factors interfere simultaneously with the variables studied (Table 3).

**Figure 3:**
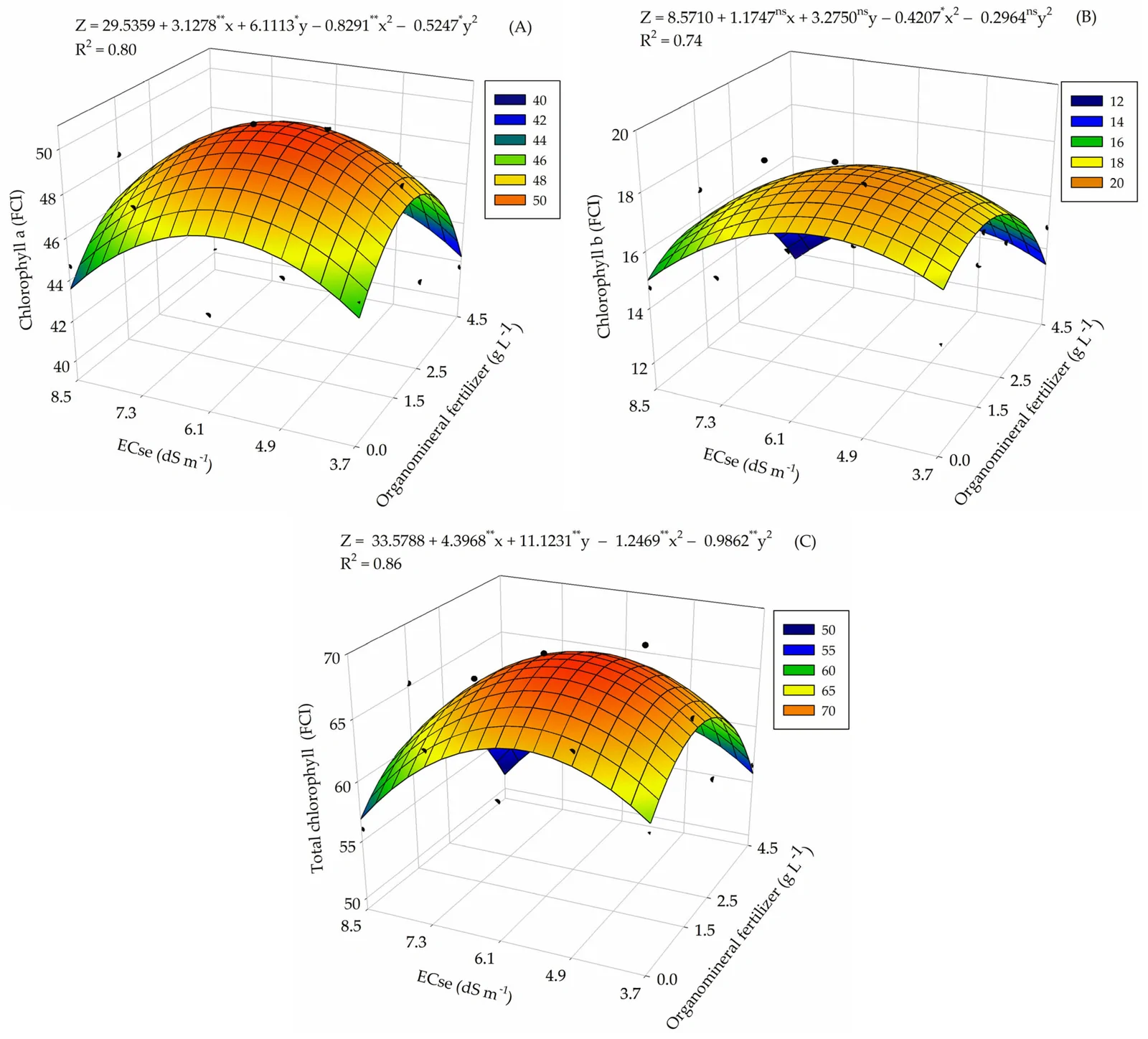
Contents of chlorophyll *a* (**A**), chlorophyll *b* (**B**) and total chlorophyll (**C**) in cotton cv. BRS Jade, as a function of the levels of electrical conductivity of the saturation extract (ECse) and concentrations of organomineral fertilizer based on mycorrhizal fungi (FMF), at 85 days after sowing. (*) significant at 0.05 probability level; (**) significant at 0.01 probability level; (ns) not significant.

Chlorophyll *a* content was higher (50.27) in plants grown in soil with an ECse of 5.8 dS m^−1^ and under an FMF concentration of 1.9 g L^−1^ (Figure 3A). On the other hand, an increase in soil salinity of up to 8.5 dS m^−1^ and the highest FMF concentration (4.5 g L^−1^) led to the lowest value (40.84) of chlorophyll *a* in cotton plants, which represents a reduction of 18.8% when compared to the maximum value obtained.

For chlorophyll *b* and total chlorophyll contents (Figure 3B,C), the maximum values (18.45 and 68.82) were obtained in plants subjected to ECse levels of 5.5 and 5.6 dS m^−1^ under FMF concentrations of 1.4 and 1.8 g ^L−1^, respectively. The lowest values (11.78 and 51.42) were observed at the highest salinity of 8.5 dS m^−1^ and an FMF concentration of 4.5 g L^−1^, reflecting reductions of 36.15 and 25.28% compared to the highest values of chlorophyll *b* and total chlorophyll in cotton plants, respectively.

### 2.4. Differential Analysis of Agronomic Traits

The interaction between the levels of electrical conductivity of the saturation extract and concentrations of organomineral fertilizer based on mycorrhizal fungi (ECse × FMF) had a significant effect on plant height and stem diameter (*p* ≤ 0.05) and on the number of leaves and leaf area (*p* ≤ 0.01) of cotton plants, cv. BRS Jade, at 85 days after sowing (Table 4).

**Figure 4:**
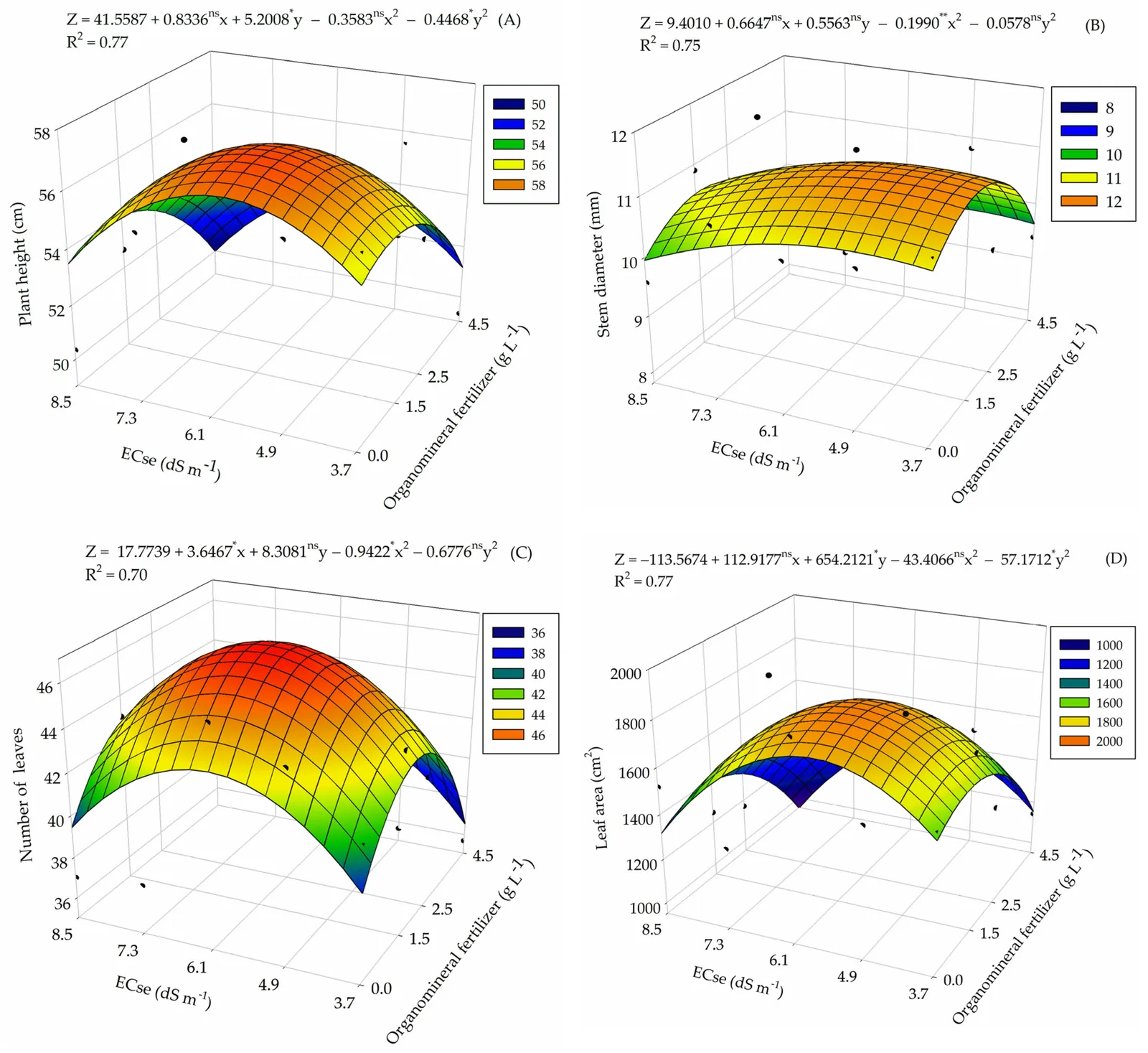
Plant height (**A**), stem diameter (**B**), number of leaves (**C**) and leaf area (**D**) of cotton cv. BRS Jade, as a function of the electrical conductivity of the saturation extract (ECse) and concentrations of organomineral fertilizer based on mycorrhizal fungi (FMF), at 85 days after sowing. (*) significant at 0.05 probability level; (**) significant at 0.01 probability level; (ns) not significant.

For plant height (Figure 4A), the highest value of 57.17 cm was observed in plants subjected to an ECse of 5.8 dS m^−1^ and an FMF concentration of 1.2 g L^−1^. On the other hand, plants cultivated under an ECse of 8.5 dS m^−1^ and an FMF concentration of 4.5 g L^−1^ obtained the minimum estimated value of 49.97 cm.

Stem diameter increased with the application of organomineral fertilizer based on mycorrhizal fungi up to a concentration of 1.7 g L^−1^ under an estimated ECse of 4.8 dS m^−1^, at which point it was equivalent to 11.28 mm; from this point on, there was a trend of inhibition, with the lowest value (8.89 mm) observed in plants under a saturation extract salinity of 8.5 dS m^−1^ and an FMF concentration of 4.5 g L^−1^ (Figure 4B).

Regarding the number of leaves (Figure 4C), the maximum estimated value (46.75) was observed in cotton plants grown under an ECse of 6.1 dS m^−1^ and an FMF concentration of 1.9 g L^−1^. The minimum value of 36.57 was reached in plants subjected to an ECse of 3.7 and an FMF concentration of 4.5 g L^−1^.

Likewise, the application of fertilizer increased leaf area up to an FMF concentration of 1.3 g L^−1^ under a saturation extract electrical conductivity of 5.7 dS m^−1^, with a maximum observed value of 1831.39 cm^2^. However, the highest concentrations of organomineral fertilizer based on mycorrhizal fungi (4.5 g L^−1^) associated with the highest ECse of 8.5 dS m^−1^ induced a reduction in leaf area, with the lowest value of 945.77 cm^2^ (Figure 4D).

The interaction between the levels of electrical conductivity of the saturation extract (ECse) and concentrations of organomineral fertilizer based on mycorrhizal fungi (FMF) had a significant effect on the leaf, stem, root, shoot and total dry mass of cotton cv. BRS Jade, at 85 days after sowing (Table 5).

**Figure 5:**
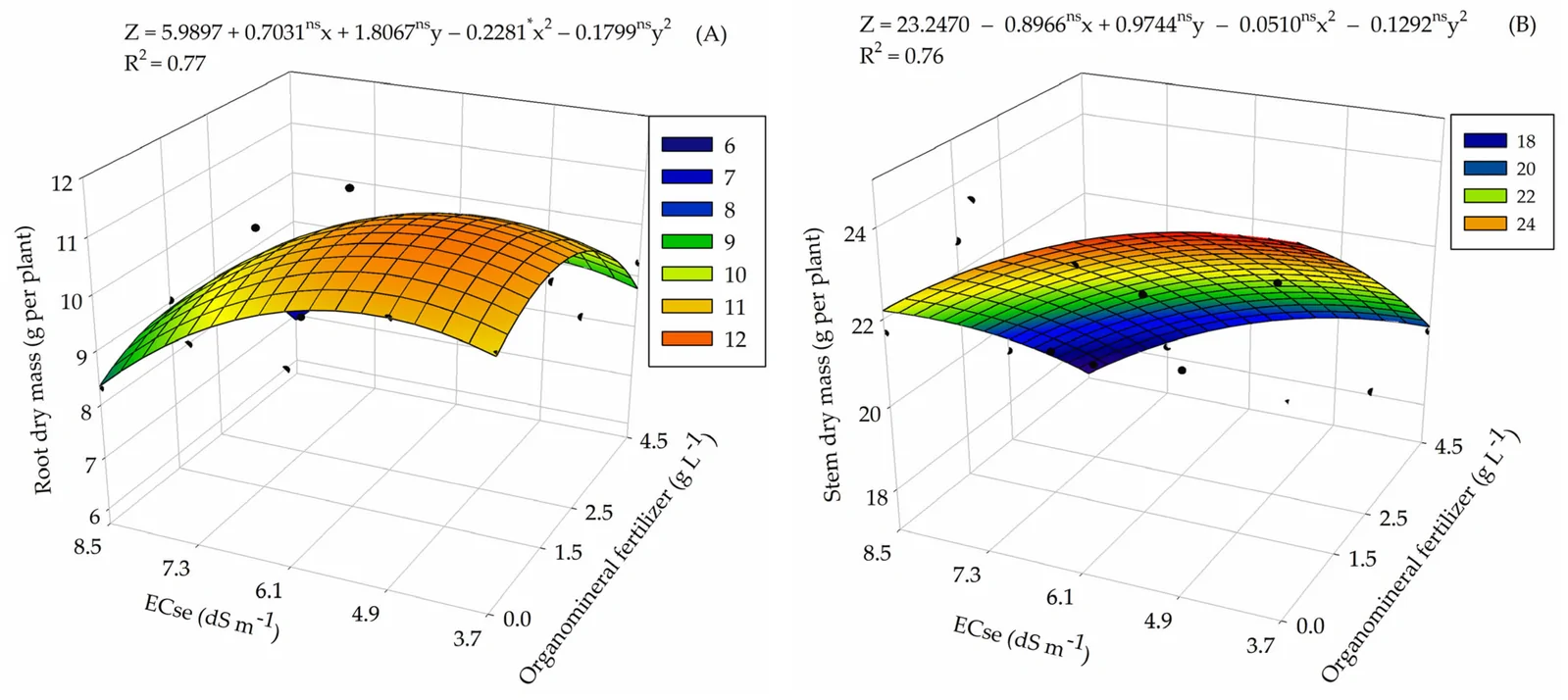

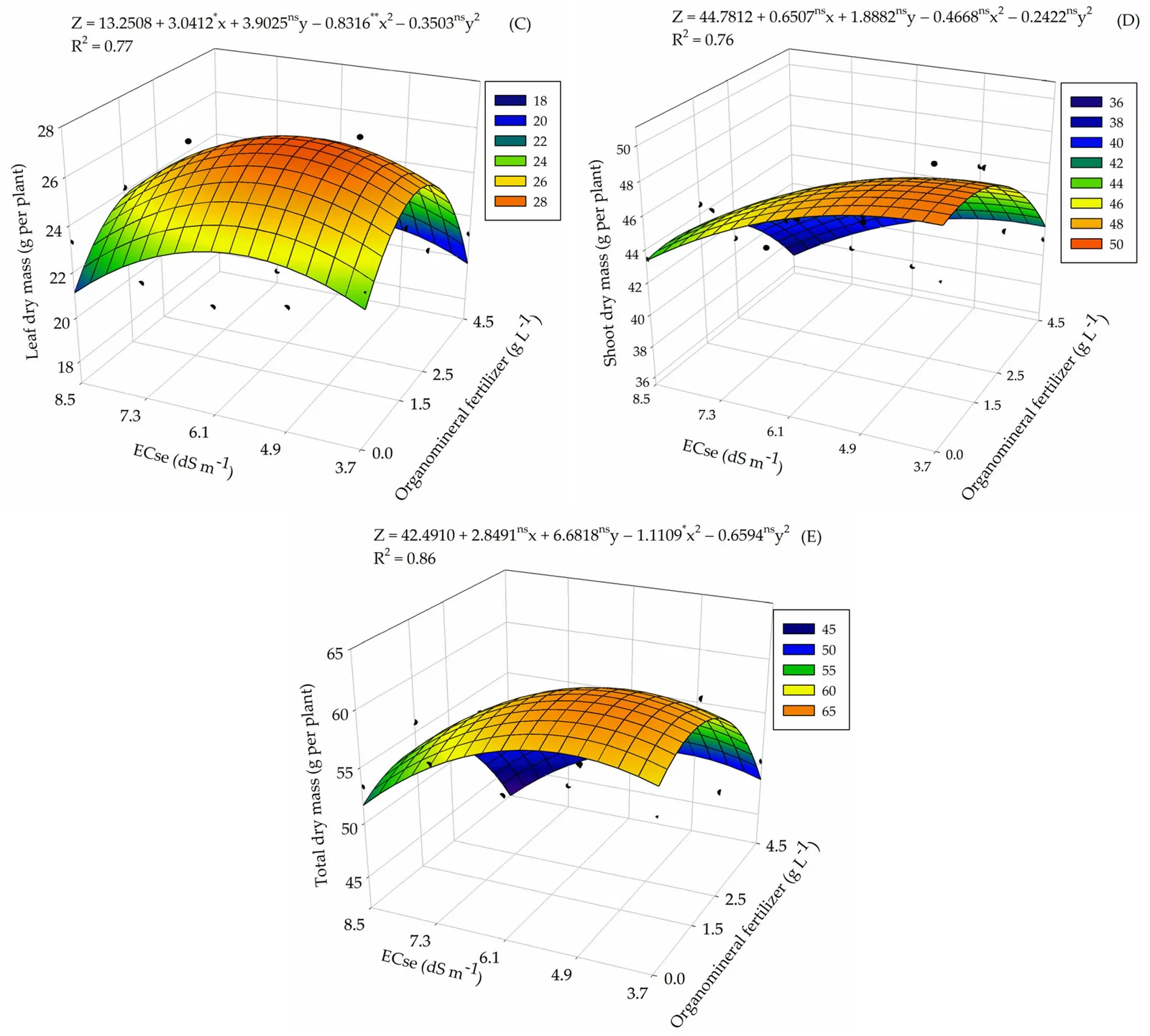
Root dry mass—RDM (**A**), stem dry mass—SDM (**B**), leaf dry mass—LDM (**C**), shoot dry mass—ShDM (**D**), and total dry mass—TDM (**E**) of cotton cv. BRS Jade as a function of the levels of electrical conductivity of the saturation extract (ECse) and concentrations of organomineral fertilizer based on mycorrhizal fungi (FMF), at 100 days after sowing. (*) significant at 0.05 probability level; (**) significant at 0.01 probability level; (ns) not significant.

Regarding biomass accumulation in cotton plants, it is possible to observe that the highest value of root dry mass (11.06 g per plant) occurred in plants subjected to an ECse of 5.0 dS m^−1^ and an FMF concentration of 1.5 g L^−1^ (Figure 5A). When the ECse level and FMF concentration were increased to their maximum levels, the lowest value was obtained (6.89 g per plant), reflecting a reduction of 37.7% compared to the highest value obtained.

For stem dry mass (Figure 5B), the concentrations of organomineral fertilizer based on mycorrhizal fungi did not promote increments, with the highest value (25.08 g per plant) in plants that did not receive FMF and at the lowest ECse of 3.7 dS m^−1^. The lowest value (17.13 g per plant) occurred at an ECse of 8.5 dS m^−1^ and an FMF concentration of 4.5 g L^−1^, which resulted in a reduction of 37.74%.

Leaf dry mass (Figure 5C) was higher (26.90 g per plant) in plants grown under an ECse of 5.6 dS m^−1^ and an FMF concentration of 1.8 g L^−1^. For shoot dry mass and total dry mass (Figure 5D,E), the highest values (48.67 and 61.21 g per plant) occurred in plants subjected to an ECse of 4.0 and 5.4 dS m^−1^ and FMF concentrations of 0.56 and 1.4 g L^−1^, respectively. Increasing ECse to 8.5 dS m^−1^ and FMF concentration to 4.5 g L^−1^ led to the lowest values (17.97, 36.81 and 41.99 g per plant), reflecting decreases of 33.19, 24.39 and 31.44% in leaf, shoot and total dry mass, respectively.

The interaction between the levels of electrical conductivity of the saturation extract and concentrations of organomineral fertilizer based on mycorrhizal fungi (ECse × FMF) had a significant effect on seed cotton mass, lint cotton mass, number of seeds and mass of seeds of colored-fiber cotton (Table 6).

**Figure 6:**
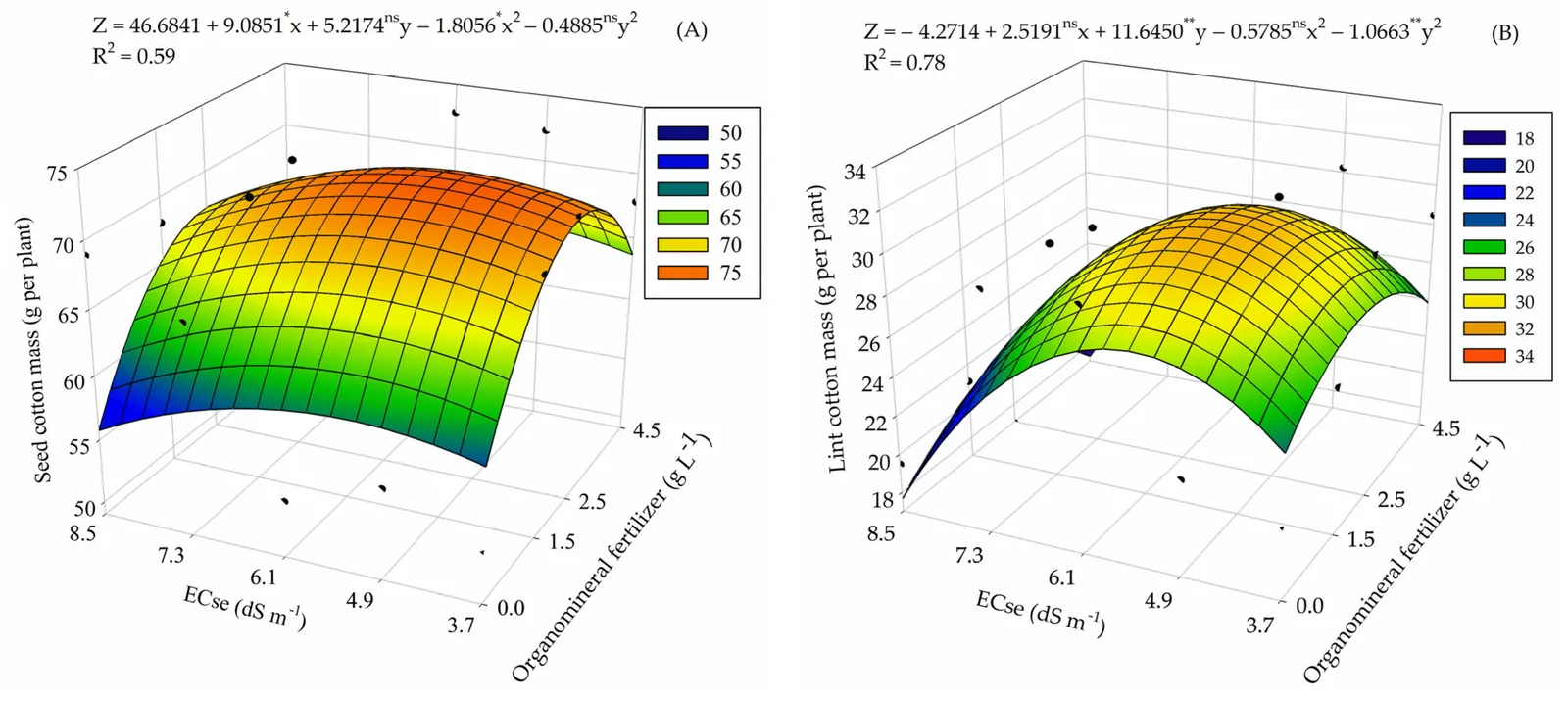

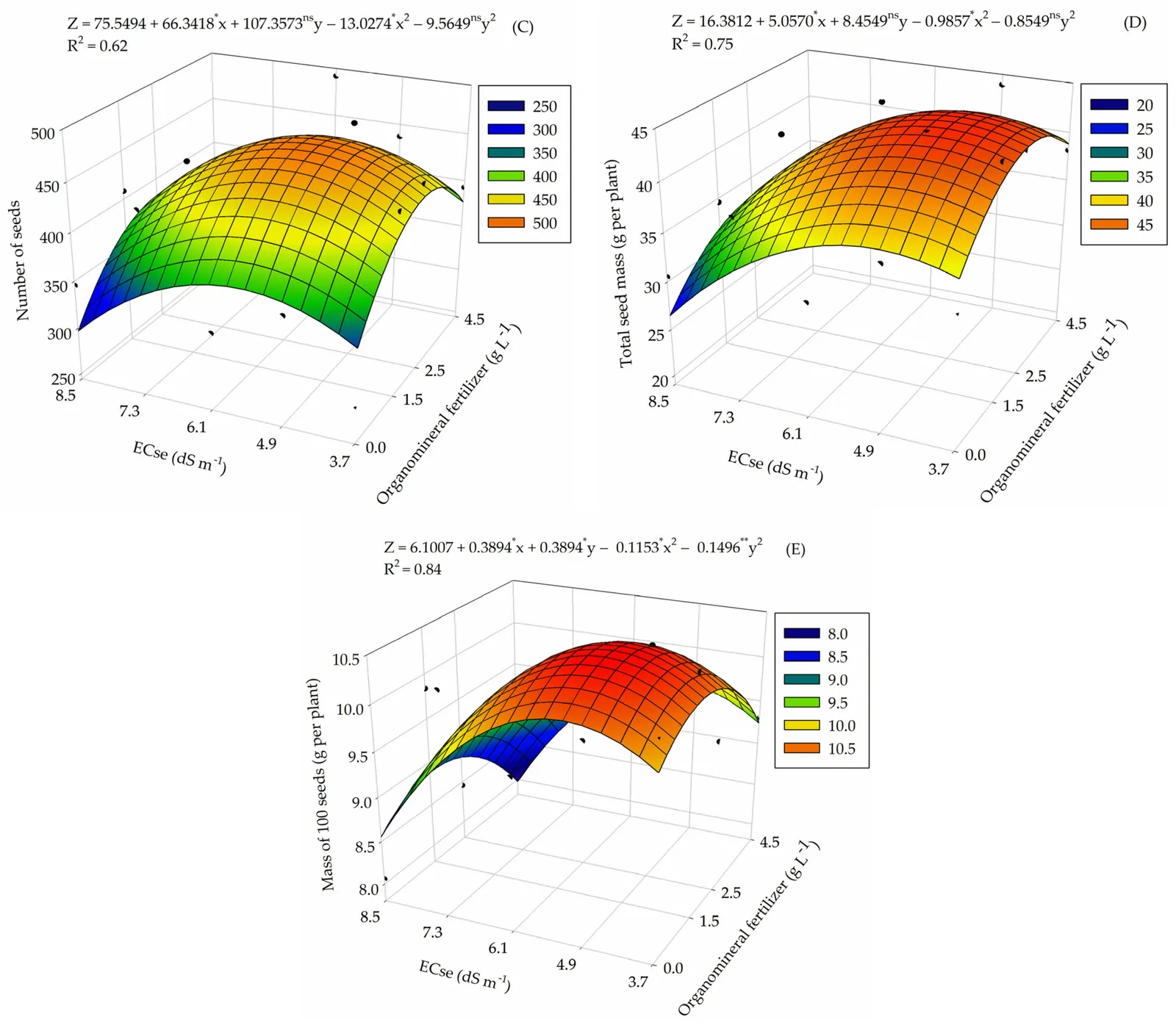
Seed cotton mass (**A**), lint cotton mass (**B**), number of seeds (**C**), total seed mass (**D**) and mass of 100 seeds (**E**) of cotton cv. BRS Jade as a function of the levels of electrical conductivity of the saturation extract and concentrations of organomineral fertilizer based on mycorrhizal fungi (FMF), at 100 days after sowing. (*) significant at 0.05 probability level; (**) significant at 0.01 probability level; (ns) not significant.

In relation to seed cotton mass, it is possible to verify that the highest value (72.02 g per plant) occurred in plants cultivated at an ECse of 5.3 dS m^−1^ and under an FMF concentration of 2.5 g L^−1^ (Figure 6A). The lowest value (55.63 g per plant) was obtained in plants subjected to an ECse of 8.5 dS m^−1^ and under no FMF application, reflecting a reduction of 22.76% when compared to the maximum values obtained.

For lint cotton mass (Figure 6B), the highest value (30.26 g per plant) was found in plants subjected to an ECse of 5.5 dS m^−1^ and an FMF concentration of 2.2 g L^−1^. On the other hand, the increase in ECse and FMF concentration to the maximum levels tested led to the lowest value (17.29 g per plant), resulting in a decrease of 42.86% compared to the maximum value obtained.

The number of seeds (Figure 6C) was highest (461 seeds per plant) in plants cultivated under an ECse of 5.6 dS m^−1^ and an FMF concentration of 2.5 g L^−1^, while the lowest value (297 seeds) occurred in plants subjected to the highest ECse and no FMF application.

Total seed mass and mass of 100 seeds (Figure 6D,E) increased as the electrical conductivity of the saturation extract increased up to 4.9 and 5.2 dS m^−1^ and the FMF concentration increased to 2.6 and 1.7 g L^−1^, respectively, reaching maximum values of 43.76 and 10.48 g per plant. The lowest values of 26.48 and 7.97 g per plant occurred at the highest soil salinity (8.5 dS m^−1^) in plants that did not receive mycorrhizal fungi and those subjected to an FMF concentration of 4.5 g L^−1^ for total seed mass and mass of 100 seeds, respectively.

### 2.5. Principal Component Analysis (PCA) Based on the Evaluated Characteristics

**Figure 7:**
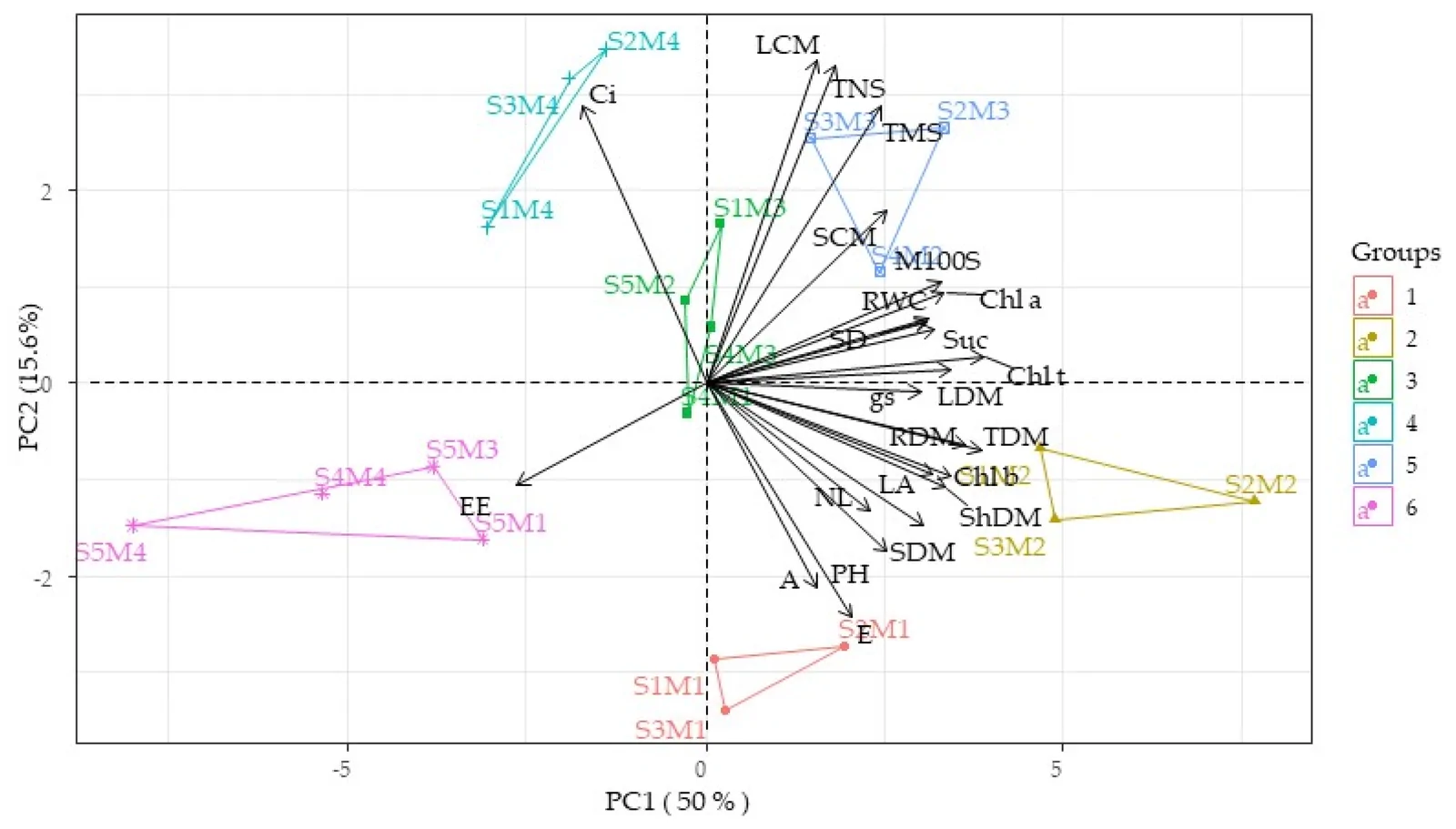
Principal component analysis (PCA) of the correlation between physiological, growth and production variables of naturally colored cotton plants, cv. BRS Jade, as a function of the interaction between electrical conductivity of the saturation extract (S1, S2, S3, S4 and S5) and concentrations of organomineral fertilizer based on mycorrhizal fungi (M1, M2, M3, M4 and M5), at 100 days after sowing.

The principal component analysis (Figure 7, Tables S1 and S2) shows the correlation between the physiological variables, growth variables and production components of cotton, with a total variability of 65.6%, with PC1 accounting for 50% of this total, especially due to the contribution of grou 2, formed by S1M2, S2M2 and S3M2 (scores 4.6892, 7.6949 and 4.8930), which have a strong correlation with biomass components (SDM, RDM, ShDM, LDM and TDM), number of leaves, leaf area and stomatal conductance. In addition, it was observed that group 1 consisted of the treatments S1M1, S2M1 and S3M1 (scores 0.1252, 1.9489 and 0.2665), which correlated with the variables CO_2_ assimilation rate, transpiration and plant height, and group 5 consisted of the treatments S2M3, S3M3 and S4M2 (scores 3.3362, 1.4696 and 2.4299), which correlated especially with the variables of the production components (LCM, TNS, TSM, SCM and M100S).

PC2 contributes 15.6% of the total variability, thanks to the contribution of group 3, formed by treatments S1M3, S4M1, S4M3 and S5M2 (scores 1.6498, −0.3149, 0.5785 and 0.4895), and group 4, consisting of S1M4, S2M4 and S3M4 (scores 1.6210, 3.4671 and 3.1714), which showed a strong correlation with the internal CO_2_ concentration (*Ci*). In addition, it is also observed that group 6, formed by treatments S4M4, S5M1, S5M3 and S5M4 (scores −1.1468, −1.6173, −0.8649 and −1.4734), had a high affinity for electrolyte leakage (EL).

## 3. Discussion

The water relationships represented by the relative water content and leaf succulence demonstrate that high soil salinity compromises the water potential of cotton plants, which can be reflected by the loss of tissue turgidity, compromising cellular processes and plant development [17]. In addition, the increase in the electrical conductivity of the saturation extract also increased the damage to leaf membranes, inducing electrolyte leakage. This is due to the fact that salt stress increases permeability and reduces the stability of membranes, especially from the accumulation of reactive oxygen species, which leads to oxidative stress [18] and may be directly associated with the accumulation of Na^+^ and Cl^−^ ions in the root zone.

On the other hand, it was possible to verify that the application of organomineral fertilizer based on mycorrhizal fungi (*Rhizophagus irregularis*) was efficient in attenuating and reducing the damage caused by soil salinity and increased the relative water content and leaf succulence in plants grown under moderate ECse levels. This effect may have occurred because the mycorrhizal-based fertilizer aids in plant osmotic adjustment and stimulates the production of osmolytes—such as soluble sugars and proline [19]—thereby reducing membrane damage (as evidenced by electrolyte leakage remaining below the 50% threshold) [20]. Furthermore, the enhanced root system development induced by mycorrhizal fungi may have helped maintain cell turgor and improve plant water status under salt stress conditions by restoring hydration and water movement through the expression of root aquaporin genes and increased water absorption capacity [21].

This reduction in cellular damage caused by salt stress may be attributed to the presence of the mycorrhizal fungus in the fertilizer composition, which confers greater plasma membrane stability to plants, reducing electrolyte leakage across the membrane; this occurs due to increased phosphorus uptake and the production of antioxidants that help protect against oxidative stress [22], a process that likely took place in the cotton plants. This phenomenon has been observed in studies showing that inoculation with mycorrhizal fungi increases antioxidant levels (peroxidase, superoxide dismutase, catalase, glutathione reductase, ascorbate peroxidase, and ascorbic acid) and reduces malondialdehyde levels in cotton plants, thereby mitigating salinity-induced oxidative stress and membrane damage [23].

The salinity of the saturation extract also promoted changes in the gas exchange of cotton cv. BRS Jade. Similar results were observed in colored-fiber cotton cv. BRS Jade, which showed that stomatal limitation caused by salt stress affected stomatal conductance, reducing transpiration and CO_2_ assimilation rate [10].

This result is supported by the reduction in osmotic potential, which limits the ability of plants to absorb water and nutrients, causing water deficit, as lower stomatal conductance may be a result of reduced soil water potential, impairing water absorption and, consequently, inducing stomatal closure [6], an adaptation mechanism that plants use to reduce water loss to the environment through transpiration and thus maintain tissue turgidity [24]. In addition, osmotic stress induced by salt stress leads to reductions in the leaf area and density of stomata and mesophyll [25], which restrict the availability and assimilation of CO_2_. In addition, stress triggers stomatal closure, reducing CO_2_ uptake and disrupting photosynthesis, ultimately leading to reductions in plant growth parameters and water use efficiency [26].

However, the use of fertilizer based on mycorrhizal fungi increases the internal CO_2_ concentration and water use efficiency of cotton plants, cv. BRS Jade, possibly due to the role of mycorrhizal fungi in improving the water absorption capacity of plants through the modulation of root hydraulic conductance, osmotic balance, and composition of osmolytes, eventually leading to dilution of the harmful effect of toxic ions, such as sodium and chlorine [13].

Salt stress inhibits the synthesis of photosynthetic pigments in cotton plants, and a marked reduction was observed under saturation with an extract salinity above 5.0 dS m^−1^. This decrease in the synthesis of photosynthetic pigments is possibly related to the increase in the activity of chlorophyllase, the enzyme responsible for chlorophyll degradation, probably caused by the greater translocation of chlorine due to the high concentration of salts in the plant or by the excess of sodium in the soil, which may have interfered with nitrogen absorption [27]. In addition, oxidative stress intensifies the effects of salinity, reducing the synthesis of chlorophylls and leading to photodegradation of the complex of pigments *a* and *b*, damaging the photosynthetic apparatus [28]. This occurred in the present study, as the increase in ECse compromised the synthesis of pigments and gas exchange of cotton plants.

However, the use of mycorrhizal fungi-based fertilizer increases the contents of chlorophyll pigments even under salt stress conditions, especially at concentrations between 1.4 and 1.8 g L^−1^, which is an indication that the increase in chlorophyll content in plants that received the application of mycorrhizae may be a mechanism to maintain photosynthetic metabolism associated with tolerance to salt stress. This is justified by the fact that mycorrhizal fungi improve the absorption of nutrients, such as nitrogen, increasing the synthesis of chlorophylls [29].

As soil salinity impaired water relations, gas exchange and pigment synthesis, it directly affected the growth and yield of cotton cv. BRS Jade. The harmful effects of high salt content in the soil on agricultural crops can be observed in several parts of the world, especially in arid and semi-arid regions [13]. The results demonstrate that the excess of salts in the soil saturation extract inhibited the growth and dry mass accumulation of cotton plants, as observed in other studies with cotton such as [5,8,10].

This is because salt stress results in water imbalance (osmotic effect) and ionic toxicity (ionic effect), paralyzing growth and affecting several physiological processes [30]. Reduced water availability limits the production of new leaves and their expansion [26] due to a reduction in soil osmotic potential, which decreases water uptake by plants, paralyzes cell expansion, and induces stomatal closure [31].

However, the application of a mycorrhizal fungus-based fertilizer reduced the deleterious effects of salt stress on the growth and dry mass accumulation of cotton plants. This likely occurred because the mycorrhizal fungi in the fertilizer, when applied to plants exposed to salinity, had the capacity to stimulate the antioxidant system to produce substances that aid in osmotic adjustment; this resulted in improved water transport and the maintenance of ionic balance (specifically the Na^+^/K^+^ ratio) via the external mycelium, thereby favoring plant development and survival under saline conditions [32].

Similarly, a study evaluating the effect of inoculation with mycorrhizal fungi on tomatoes observed a considerable increase in shoot and root biomass—yielding gains of 43.8% and 92.3%, respectively—compared to non-inoculated plants under salt stress conditions [14]. Research has shown that the use of mycorrhizal fungi mitigates the effects of salt stress on the growth and biomass production of shoots and roots in soybean plants [33]; this supports our findings, indicating that the inclusion of mycorrhizal fungi in the fertilizer composition can enhance the growth of BRS Jade cotton plants cultivated in saline soil.

The production components of cotton are reduced under saline conditions, which is mainly due to the reduced emergence of flower buds and the fall of flowers and bolls resulting from the osmotic and ionic effects of salinity [9,10], as observed in the present study using soil with a high salinity level. However, our findings show that plants irrigated with up to 5.0 dS m^−1^, associated with the application of fertilizer based on mycorrhizal fungi at a concentration of 2.0 g L^−1^, had higher values for these parameters.

This behavior likely occurred because the fertilizer stimulated root elongation, thereby expanding the plants’ water absorption area to mitigate cellular water deficit; this inhibited stomatal closure, reduced chloroplast damage, and improved light energy use efficiency [22], consequently increasing the accumulation of photoassimilates and yield components.

The behavior observed in the principal component analysis highlights the positive effect of the mycorrhizal fungus-based fertilizer—particularly on cotton growth and yield—at a concentration of 2.5 g L^−1^ under moderate salinity conditions; this occurs because mycorrhizae help maintain chlorophyll content and improve nutrient uptake—facilitated by greater root expansion—thereby increasing the crop’s tolerance to the effects of salt stress [34].

It was also observed that the negative effects of higher soil electrical conductivity levels (above 5.0 dS m^−1^) were intensified by the maximum concentration (4.5 g L^−1^) of the tested fertilizer; this reduced gas exchange—specifically transpiration, the CO_2_ assimilation rate, and intrinsic carboxylation efficiency—and was associated with a reduction in photosynthetic pigments, ultimately resulting in losses in cotton plant growth and yield. This effect may be associated with increased salt accumulation in the cotton plant root zone, which limits the plant’s ability to absorb water and nutrients due to a reduction in soil osmotic potential; this induces water and nutritional deficits, thereby compromising physiological processes, growth, and crop yield [35]. Furthermore, ionic effects (Na^+^ and CL^−^) trigger a series of disturbances—such as oxidative stress—that impair the photosynthetic apparatus through enzyme inhibition and denaturation [36], potentially accounting for the losses observed in the study.

## 4. Materials and Methods

### 4.1. Experimental Site

**Figure 8:**
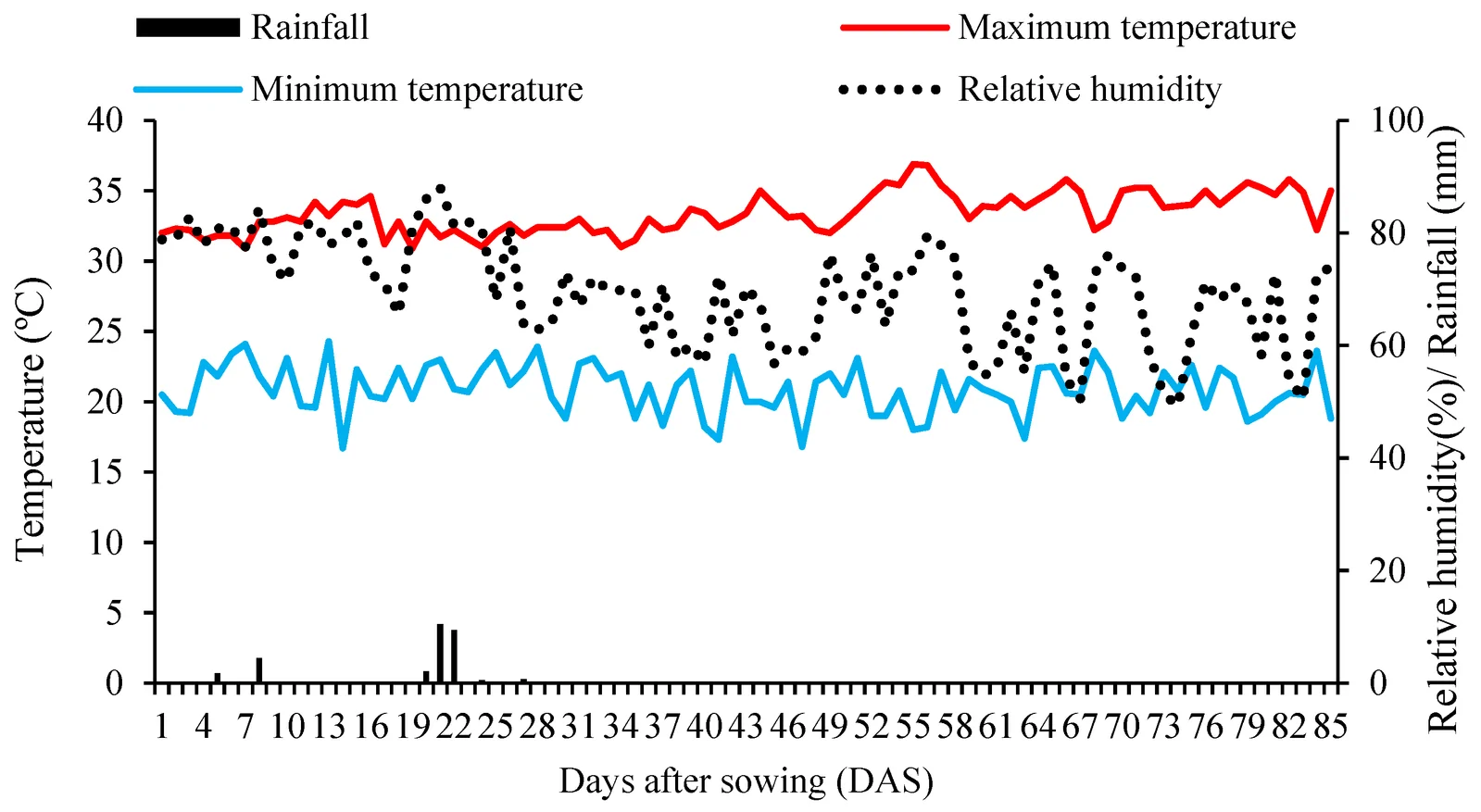
Data on maximum and minimum temperature (°C), relative humidity (%) and rainfall during the experimental period. Source: INMMET [38].

The experiment was conducted from June to September 2024 under outdoor lysimeter experiment under ambient climatic conditions at the Center for Sciences and Agri-Food Technology (CCTA) at the Federal University of Campina Grande (UFCG) in Pombal, PB, Brazil, at the geographic coordinates 6°47′20″ S latitude and 37°48′01″ W longitude, at altitude of 194 m, under a BSh climate, that is, hot and dry semi-arid, according to Koppen’s classification [37]. Climatic data on temperature and humidity are described in Figure 8.

### 4.2. Statistical Design and Treatments

The experimental design was randomized blocks, in a 5 × 4 factorial scheme, referring to five levels of electrical conductivity of the soil saturation extract (ECse) (S1 = 3.7; S2 = 4.9; S3 = 6.1; S4 = 7.3 and S5 = 8.5 dS m^−1^) and four concentration of mycorrhizal fungus-based fertilizer (M1 = 0 (control); M2 = 1.5; M3 = 2.5 and M4 = 4.5 g L^−1^), with three replicates, totaling 60 experimental units. The levels of electrical conductivity of the saturation extract were established according to [39], considering the salinity threshold level of the saturation extract for white-fiber cotton. The concentrations of the fertilizer containing mycorrhizal fungi were based on the manufacturer’s recommendation.

### 4.3. Experiment Conduction and Treatment Application

Cotton plants were grown in plastic pots adapted as drainage lysimeters with 20 L capacity (dimensions: 325 × 340 mm diameter x height) filled with a 500 g layer of crushed stone No. 0 on top of a polypropylene screen to avoid drain clogging with the soil material. A 15 mm diameter hose was installed at the base of each container as a drain and coupled to a plastic container (2 L) to collect the drained water.

Then, the pots were filled with 22 kg of soil classified as *Neossolo Regolítico* (Entisol) with sandy loam texture, properly pounded to break up clods and homogenized; it was collected from the 0–20 cm layer in the experimental area of the ‘Rolando Enrique Rivas Castellón’ Farm, belonging to the CCTA/UFCG, in São Domingos, PB. The soil was subjected to prior analysis, and its physical and chemical characteristics (Table 7) were determined according to the methodology [40].

**Table 7:** Chemical and physical characteristics of the soil used in the experiment before application of the treatments.

Chemical characteristics								
pH (H_2_O)	OM	P	K^+^	Na^+^	Ca^2+^	Mg^2+^	Al^3+^	H^+^
(1:2.5)	g kg^−1^	(mg kg^−1^)	cmol_c_ kg^−1^					
6.01	0.21	0.53	0.12	0.05	3.00	2.44	0.00	0.69
Chemical characteristics				Physical characteristics				
EC_se_	CEC	SAR_se_	ESP	Particle-size fraction (g kg^−1^)			Moisture (dag kg^−1^)	
(dS m^−1^)	cmol_c_ kg^−1^	(mmol L^−1^)^0.5^	%	Sand	Silt	Clay	33.42 kPa ^1^	1519.5 kPa ^2^
0.71	6.25	0.61	0.80	756.50	200.10	43.40	33.57	5.01

pH—hydrogen potential, OM—organic matter: Walkley–Black Wet Digestion; Ca^2+^ and Mg^2+^ extracted with 1 M KCl at pH 7.0; Na^+^ and K^+^ extracted with 1 M NH_4_OAc at pH 7.0; Al^3+^ + H^+^ extracted with 0.5 M CaOAc at pH 7.0; EC_se_—electrical conductivity of saturated paste extract; CEC—cation exchange capacity; SAR_se_—sodium adsorption ratio of saturated paste extract; ESP—exchangeable sodium percentage. Superscripts ^1^ and ^2^ refer to moisture contents in the soil corresponding to field capacity and permanent wilting point, respectively.

After filling the lysimeters, the soil was salinized by dissolving non-iodized NaCl applied via irrigation in order to obtain ECse levels of 3.7, 4.9, 6.1, 7.3 and 8.5 dS m^−1^. The soil used at the 3.7 dS m^−1^ level is classified as non-saline since the electrical conductivity of the saturation extract is less than 4.0 dS m^−1^ [41]. The amount of salt to be incorporated into the soil was determined through a pre-test, with doses calculated according to Rhoades [42], using Equation (1).

(1) $$C=640\left(ECse-ECsei\right)\times DSW$$

where

C—Quantity of salts to be added (mg L^−1^).

ECse—Desired value of electrical conductivity of soil saturation extract (dS m^−1^).

ECsei—Initial value of the electrical conductivity of the soil saturation extract (dS m^−1^).

DSW—Dry soil weight (kg).

Prior to planting, the moisture content was raised to the level corresponding to the maximum water retention capacity, using water with low electrical conductivity (ECw = 0.3 dS m^−1^).

Irrigation was performed daily in the late afternoon, with the volume of water applied according to the water balance method [43], accounting for the amounts applied and drained, and employing a leaching fraction of 0.10. After drainage, the leachate volume was applied to the soil, according to each treatment, in order to maintain the levels of ECse.

Every 15 days, the electrical conductivity (EC) of the soil saturation extract was analyzed by saturating the soil and subsequently measuring the EC of the drained water using a benchtop conductivity meter (Tecnopon model MCA-150). Following the readings, the soil EC was adjusted to the levels of the established treatments whenever necessary.

Sowing was carried out using 3 seeds of the colored cotton cultivar ‘BRS Jade’ distributed equidistantly in each lysimeter. At 10 days after sowing (DAS), the plants were thinned, and only one was kept in each lysimeter. Top-dressing fertilization began at 15 DAS, using urea (45% N), monoammonium phosphate (9% N and 48% P_2_O_5_) and potassium chloride (60% K_2_O), respectively, as sources of nitrogen, phosphorus and potassium, according to the recommendation of [44].

At 20 DAS, the mycorrhizal fungi were applied according to the proposed treatments (0, 1.5, 2.5 and 4.5 g L^−1^), considering the manufacturer’s recommendation. The fertilizer was added in a single application to the drainage lysimeters, diluted in 67 mL of water (H_2_O) for each treatment. The product used was Phosmyc^®^ (TopBio Sistemas Biológicos (Tibau, Brazil)), an organomineral fertilizer based on the fungus *Rhizophagus irregularis*—which forms arbuscular mycorrhizae—with a concentration of 1 × 10^5^ spores/kg plus propagules, 43.0% *w*/*w* organic carbon, 7.0% *w*/*w* N, 3.0% *w*/*w* P_2_O_5_, and 3.0% *w*/*w* Si.

### 4.4. Variables Analyzed

At 85 DAS, the relative water content (RWC) in the leaf blade, electrolyte leakage (EL) and leaf succulence (SUC) were evaluated. RWC was determined from the collection of three fully expanded leaves in the middle third of the plant. Subsequently, 4 disks with an area of 12 mm were collected, and the fresh mass (FM) was determined; then, the samples were placed in plastic bags, immersed in distilled water and stored for 24 h. After this period, excess water was removed with the help of paper towels to determine the turgid mass (TM); subsequently, the samples were taken to an oven with air circulation (65° ± 3 °C) and kept until they reached a constant mass to obtain the dry mass (DM). RWC values were measured according to the methodology adapted from [45].

Electrolyte leakage in the leaf blade was obtained according to Scotti-Campos [46]. Leaves were collected from the middle third of the plant, and 4 leaf disks of 12 mm were removed and placed in beakers with 50 mL of distilled water, which were closed with aluminum foil. The beakers were maintained at a temperature of 25 °C for 24 h, after which the initial electrical conductivity (Ci) was measured. Subsequently, the beakers were taken to the incubator, with forced-air ventilation and subjected to a temperature of 80 °C for approximately 90 min, after which the final electrical conductivity (Cf) was measured, thus obtaining the percentage of electrolyte leakage. Leaf succulence (SUC) was determined at 90 DAS, according to the methodology proposed by Mantovani [47].

Gas exchange was evaluated at 85 DAS, based on the following measurements: stomatal conductance—*gs* (mmol H_2_O m^−2^ s^−1^), transpiration—*E* (mmol H_2_O m^−2^ s^−1^), CO_2_ assimilation rate—*A* (μmol CO_2_ m^−2^ s^−1^) and internal CO_2_ concentration—*Ci* (μmol mol^−1^). These data were then used to estimate the instantaneous water use efficiency (*WUEi*) (*A*/*E*) [(μmol CO_2_ m^−2^ s^−1^) (mmol H_2_O m^−2^ s^−1^)^−1^]. Evaluations were carried out with a plant gas exchange meter containing an infrared gas analyzer (IRGA) (Infra Red Gas Analyser, model CIRAS-3, PP Systems, Amesbury, MA, United States (USA)). Readings were taken on the third fully expanded leaf counted from the apical bud at 07:00 a.m. under natural conditions for air temperature and CO_2_ concentrations, using an artificial radiation source of 1200 μmol m^−2^ s^−1^.

For photosynthetic pigments, the contents of chlorophyll *a*, chlorophyll *b* and total chlorophyll were estimated using a non-destructive method, evaluating fully expanded leaves in the middle third of the plant, with the aid of a portable electronic chlorophyll meter (ClorofiLOG^®^, model CFL2060, Porto Alegre, Brazil).

Cotton growth was estimated at 85 DAS, based on the following parameters: number of leaves, determined by counting mature leaves longer than 3 cm; plant height, measured from the collar to the insertion of the apical meristem, with a ruler graduated in cm; and stem diameter, measured at 2 cm from the ground using a digital caliper, with readings in mm. Leaf area (LA) was determined according to Grimes and Carter [48] by measuring the length of the main vein of each leaf of the plants to obtain the LA value, considering fully expanded leaves for the measurement.

At 100 DAS, three plants per treatment were collected, and their roots, stems and leaves were separated, packed in paper bags and dried in an air circulation oven at a temperature of 65 °C until reaching constant weight. After drying, the material was weighed on a semi-analytical scale with a precision of 0.001 g to obtain stem dry mass (SDM, g per plant), leaf dry mass (LDM, g per plant), and root dry mass (RDM, g per plant). SDM and LDM were summed to obtain shoot dry mass (ShDM, g per plant), and all fractionated parts were summed to obtain the total dry mass (TDM, g per plant).

In the same period, the production components were quantified as follows: seed cotton mass (SCM, g per plant), obtained by weighing on a semi-analytical scale with a precision of 0.001 g; lint cotton mass (LCM, g per plant), obtained by weighing the fibers after delinting; total number of seeds (TNS) obtained by counting the seeds; total seed mass (TSM, g per plant), obtained by weighing the seeds on a semi-analytical scale; and mass of 100 seeds (M100S), determined by weighing 100 seeds.

### 4.5. Statistical Analysis

The data were subjected to the distribution normality test (Shapiro–Wilk), and subsequently, analysis of variance (*p* ≤ 0.05) was performed using the F test. In cases of significance, polynomial regression analysis was performed using the statistical software SISVAR-ESAL, version 5.7 [49]. In cases of significance for the interaction between factors, the SigmaPlot^®^ 12.5 software was used to create the response surfaces. The data were subjected to principal component analysis (PCA) using a correlation matrix. Component selection followed the Kaiser criterion, retaining those with eigenvalues greater than 1. Variables with eigenvectors (factor loadings) exceeding 0.5 in absolute value were considered relevant to the formation of the components. Observations were classified via cluster analysis, using Gower distance to construct the dissimilarity matrix. The number of clusters was determined using the Average Silhouette Method, aiming to maximize intra-cluster similarity and inter-cluster dissimilarity. Statistical analyses were performed using the R software, version 4.4.0 [50].

## 5. Conclusions

Based on the results presented, it is concluded that soil salinity negatively affects the growth and productivity of the cotton cultivar BRS Jade due to water imbalance, ionic toxicity (Na^+^ and Cl^−^), and reduced photosynthetic capacity. However, under the experimental conditions studied, the application of an organomineral fertilizer based on mycorrhizal fungi proved to be an effective strategy for mitigating these deleterious effects under moderate salinity conditions; this indicates that its use may constitute a promising agronomic practice for enhancing cotton tolerance to salt stress, thereby promoting beneficial effects on growth and productivity in arid and semi-arid regions.

## Figures and Tables

**Table 1:** Summary of the analysis of variance for the relative water content (RWC), electrolyte leakage (EL) and leaf succulence (SUC) of cotton cv. BRS Jade cultivated under different levels of electrical conductivity of the saturation extract (ECse) and concentrations of organomineral fertilizer based on mycorrhizal fungi (FMF), at 85 days after sowing.

Sources of Variation	DF	Mean Squares		
		RWC	EL	SUC
Soil salinity (ECse)	4	126.80 **	244.08 **	0.00004 **
Linear regression	1	372.46 **	940.13 **	0.000059 **
Quadratic regression	1	89.52 **	25.65 ^ns^	0.000077 **
Fertilizer organo-mineral (FMF)	3	138.05 **	279.00 **	0.00006 **
Linear regression	1	47.46 **	12.37 ^ns^	0.000029 **
Quadratic regression	1	365.33 **	717.53 **	0.00011 **
Interaction (ECse × FMF)	12	38.06 **	40.51 **	0.000005 **
Blocks	2	4.73 ^ns^	2.46 ^ns^	0.0000004 ^ns^
Residual	38	4.12	9.27	0.00000008
CV (%)		5.81	10.66	13.64

DF—degrees of freedom; CV (%)—coefficient of variation; (**) significant at 0.01 probability level; (^ns^) not significant.

**Table 2:** Summary of the analysis of variance for stomatal conductance (*gs*), transpiration (*E*), internal CO_2_ concentration (*Ci*), CO_2_ assimilation rate (*A*) and instantaneous water use efficiency (*WUEi*) of cotton cv. BRS Jade cultivated under different levels of electrical conductivity of the saturation extract (ECse) and concentrations of organomineral fertilizer based on mycorrhizal fungi (FMF), at 85 days after sowing.

Sources of Variation	DF	Mean Squares				
		*gs*	*E*	*Ci*	*A*	*WUEi*
Soil salinity (ECse)	4	11,604.56 **	0.96 ^ns^	356.61 ^ns^	41.09 **	4.09 ^ns^
Linear regression	1	44,352.08 **	0.24 ^ns^	1190.70 *	161.29 **	11.47 ^ns^
Quadratic regression	1	20.72 ^ns^	2.10 ^ns^	184.38 ^ns^	1.10 ^ns^	4.34 ^ns^
Organomineral fertilizer (FMF)	3	21,001.16 **	19.73 **	7551.35 **	250.29 **	15.54 **
Linear regression	1	29,760.48 **	58.61 **	22,481.36 **	727.31 **	12.62 **
Quadratic regression	1	41,396.27 **	0.04 ^ns^	156.82 ^ns^	1.32 ^ns^	32.82 **
Interaction (ECse × FMF)	12	6472.45 **	2.58 **	825.81 **	52.58 **	4.01 *
Blocks	2	1009.27 ^ns^	0.74 ^ns^	4315.32 **	29.62 *	0.63 ^ns^
Residual	38	1159.00	0.42	260.25	9.13	1.73
CV (%)		20.11	14.77	19.37	12.04	21.37

DF—degrees of freedom; CV (%)—coefficient of variation; (*) significant at 0.05 probability level; (**) significant at 0.01 probability level; (ns) not significant.

**Table 3:** Summary of the analysis of variance for the contents of chlorophyll *a* (Chl *a*), chlorophyll *b* (Chl *b*) and total chlorophyll (Chl T) of cotton cv. BRS Jade cultivated under different levels of electrical conductivity of the saturation extract (ECse) and concentrations of organomineral fertilizer based on mycorrhizal fungi (FMF), at 85 days after sowing.

Sources of Variation	DF	Mean Squares		
		Chl *a*	Chl *b*	Chl T
Soil salinity (ECse)	4	42.23 **	17.32 **	148.29 **
Linear regression	1	14.53 ^ns^	20.13 *	142.79 **
Quadratic regression	1	95.93 **	30.56 **	338.87 **
Organomineral fertilizer (FMF)	3	108.39 **	71.44 **	338.74 **
Linear regression	1	58.34 **	92.41 **	259.10 **
Quadratic regression	1	260.83 **	81.20 **	663.10 **
Interaction (ECse × FMF)	12	13.04 **	7.48 *	30.62 *
Blocks	2	21.46 *	4.37 ^ns^	26.27 ^ns^
Residual	38	4.17	3.87	8.93
CV (%)		4.39	12.19	4.80

DF—degrees of freedom; CV (%)—coefficient of variation; (*) significant at 0.05 probability level; (**) significant at 0.01 probability level; (ns) not significant.

**Table 4:** Summary of the analysis of variance for plant height (PH), stem diameter (SD), number of leaves (NL) and leaf area (LA) of cotton cv. BRS Jade cultivated under different levels of electrical conductivity of the saturation extract (ECse) and concentrations of organomineral fertilizer based on mycorrhizal fungi (FMF), at 85 days after sowing.

Sources of Variation	DF	Mean Squares			
		PH	SD	NL	LA
Soil salinity (ECse)	4	23.93 **	1.29 ^ns^	43.81 **	370,324.75 **
Linear regression	1	10.80 ^ns^	3.81 ^ns^	0.30 ^ns^	323,634.41 **
Quadratic regression	1	15.74 **	1.98 ^ns^	160.09 **	1,138,652.88 **
Organomineral fertilizer (FMF)	3	50.91 **	8.69 **	136.22 **	826,523.07 **
Linear regression	1	96.33 **	8.84 **	58.08 *	1,197,517.76 **
Quadratic regression	1	56.07 **	16.02 **	336.07 **	877,290.08 **
Interaction (ECse × FMF)	12	11.80 *	1.56 *	43.15 **	134,029.48 **
Blocks	2	1.52 ^ns^	0.53 ^ns^	10.62 ^ns^	114,380.54 *
Residual	38	4.41	0.59	9.70	26,140.97
CV (%)		3.85	7.35	7.37	10.69

DF—degrees of freedom; CV (%)—coefficient of variation; (*) significant at 0.05 probability level; (**) significant at 0.01 probability level; (ns) not significant.

**Table 5:** Summary of the analysis of variance for leaf dry mass (LDM), stem dry mass (SDM), root dry mass (RDM), shoot dry mass (ShDM) and total dry mass (TDM) of cotton cv. BRS Jade cultivated under different levels of electrical conductivity of the saturation extract (ECse) and concentrations of organomineral fertilizer based on mycorrhizal fungi (FMF), at 100 days after sowing.

Sources of Variation	DF	Mean Squares				
		LDM	SDM	RDM	ShDM	TDM
Soil salinity (ECse)	4	22.60 **	28.30 **	10.49 **	107.72 **	162.60 **
Linear regression	1	23.87 *	62.71 **	26.06 **	196.53 **	320.75 **
Quadratic regression	1	42.86 **	5.78 ^ns^	11.28 **	20.45 ^ns^	151.52 **
Organomineral fertilizer (FMF)	3	134.17 **	89.88 **	19.69 **	164.06 **	559.65 **
Linear regression	1	85.86 **	221.83 **	19.56 **	352.39 **	816.98 **
Quadratic regression	1	275.25 **	6.82 ^ns^	23.52 **	111.58 **	578.52 **
Interaction (ECse × FMF)	12	14.31 **	7.33 *	2.09 *	16.21 *	19.73 *
Blocks	2	3.74 ^ns^	0.85 ^ns^	2.40 ^ns^	12.07 ^ns^	14.98 ^ns^
Residual	38	4.18	4.03	0.91	7.58	9.27
CV (%)		8.70	9.24	9.87	6.17	5.55

DF—degrees of freedom; CV (%)—coefficient of variation; (*) significant at 0.05 probability level; (**) significant at 0.01 probability level; (ns) not significant.

**Table 6:** Summary of the analysis of variance for seed cotton mass (SCM), lint cotton mass (LCM), total number of seeds (TNS), total seed mass (TSM) and mass of 100 seeds (M100S) of cotton cv. BRS Jade cultivated under different levels of electrical conductivity of the saturation extract (ECse) and concentrations of organomineral fertilizer based on mycorrhizal fungi (FMF), at 100 days after sowing.

Sources of Variation	DF	Mean Squares				
		SCM	LCM	TNS	TSM	M100S
Soil salinity (ECse)	4	54.93 ^ns^	194.66 **	11,929.18 **	244.88 **	5.09 **
Linear regression	1	95.03 ^ns^	321.57 **	15,052.80 **	674.08 **	12.04 **
Quadratic regression	1	83.06 ^ns^	396.009 **	31,872.60 **	254.56 **	7.82 **
Organomineral fertilizer (FMF)	3	417.37 **	38.88 **	21,818.55 **	130.41 **	2.66 **
Linear regression	1	181.62 **	0.60 ^ns^	11,150.80 **	61.59 *	2.66 **
Quadratic regression	1	1011.55 **	114.82 **	52,866.02 **	317.56 **	5.24 **
Interaction (ECse × FMF)	12	213.29 **	38.64 **	14,388.27 **	78.85 **	0.78 *
Blocks	2	1.90 ^ns^	9.06 ^ns^	465.62 ^ns^	2.72 ^ns^	0.32 ^ns^
Residual	38	22.63	4.63	1033.58	9.17	0.32
CV (%)		7.29	8.54	8.15	8.11	5.87

DF—degrees of freedom; CV (%)—coefficient of variation; (*) significant at 0.05 probability level; (**) significant at 0.01 probability level; (ns) not significant.

## Data Availability

The data presented in this study are available on request from the corresponding author. The data are not publicly available due to privacy and ethical restrictions.

## Supplementary Materials

The following supporting information can be downloaded at: https://www.mdpi.com/article/10.3390/plants15213247/s1, Table S1. Vector values from the principal component analysis based on the performance of the analyzed variables and Table S2. Principal component analysis score values based on treatment performance.
