# Supplementary material for: Mycorrhizal Fungus-Based Fertilizer Mitigates Salt Stress Damage on Colored-Fiber Cotton in Semi-Arid Salinized Soil of Brazil
Source: Plants (Basel). 2026 Oct 22;15(21):3247. doi: 10.3390/plants15213247 (PMC13765512; doi:10.3390/plants15213247)
Supplement: Supplementary file 1 [file plants-15-03247-s001.zip › plants-4580981-supplementary.pdf]

Supplementary data

Table S1. Vector values from the principal component analysis based on the performance of the analyzed variables

Variables	Vetor CP1	Vetor CP2
PH	-0.6157	-0.4252
SD	-0.7524	0.1567
NF	-0.5604	-0.3200
Ci	0.4160	0.7022
Gs	-0.7322	-0.0210
A	-0.3767	-0.5136
E	-0.4941	-0.5920
LDM	-0.8324	0.0363
SDM	-0.7398	-0.3590
RDM	-0.8859	-0.1539
ShDM	-0.8146	-0.2599
TDM	-0.9370	-0.1694
LA	-0.7734	-0.2300
SCM	-0.3748	0.8183
PB	-0.7794	0.1380
Chlo a	-0.8111	0.2286
Chlo b	-0.8356	-0.2359
Chlo t	-0.9430	0.0688
RWC	-0.7563	0.1621
EL	0.6398	-0.2550
Suc	-0.8008	0.1744
LCM	-0.6136	0.4364
TNS	-0.4376	0.8036
TSM	-0.5971	0.6992
M100S	-0.8009	0.2574

Legend: Relative water content – RWC, electrolyte leakage – EL, leaf succulence – SUC, Stomatal conductance – *gs*, transpiration – *E*, internal CO_2 concentration – *Ci*, CO_2 assimilation rate – *A*, instantaneous water use efficiency – *WUEi,* chlorophyll *a* -Chlo a, chlorophyll *b* – Chlo b, total chlorophyll – Chlo t, Plant height - PH, stem diameter - SD, number of leaves – NL, leaf area – LA, Root dry mass - RDM, stem dry mass - SDM, leaf dry mass - LDM, shoot dry mass – ShDM, total dry mass – TDM, seed cotton mass - SCM, lint cotton mass - LCM, total number of seeds - TNS, total seed mass - TSM and mass of 100 seeds - M100S.

Table S2. Principal component analysis score values based on treatment performance

Treatment	**Score PC1**	**Score PC2**	**Groups**
S1M1	0.12522657	-2.8534843	1
S1M2	4.68926368	-0.6684857	2
S1M3	0.20969288	1.6498629	3
S1M4	-3.03370776	1.6210661	4
S2M1	1.94890582	-2.7220448	1
S2M2	7.69494417	-1.2214501	2
S2M3	3.33223446	2.6537419	5
S2M4	-1.40343749	3.4671294	4
S3M1	0.26651599	-3.3931455	1
S3M2	4.89301586	-1.4204036	2
S3M3	1.46965254	2.5475095	5
S3M4	-1.89607790	3.1714147	4
S4M1	-0.25480182	-0.3149840	3
S4M2	2.42990002	1.1578418	5
S4M3	0.07111968	0.5785865	3
S4M4	-5.34974153	-1.1468982	6
S5M1	-3.10106845	-1.6173893	6
S5M2	-0.29267352	0.8495362	3
S5M3	-3.79306507	-0.8649457	6
S5M4	-8.00589813	-1.4734577	6

Legend: Electrical conductivity of the saturation extract (S1, S2, S3, S4 and S5) and concentrations of organomineral fertilizer based on mycorrhizal fungi (M1, M2, M3, M4 and M5),
